# Theoretical Insights into Hydrogen Production from Formic Acid Catalyzed by Pt-Group Single-Atom Catalysts

**DOI:** 10.3390/ma18102328

**Published:** 2025-05-16

**Authors:** Tao Jin, Sen Liang, Jiahao Zhang, Yaru Li, Yukun Bai, Hangjin Wu, Ihar Razanau, Kunming Pan, Fang Wang

**Affiliations:** 1 Longmen Laboratory, Luoyang 471000, Chinawangfang1116@163.com (F.W.); 2 School of Materials Science and Engineering, Henan University of Science and Technology, Luoyang 471000, China; 3 Scientific-Practical Materials Research Centre, National Academy of Sciences of Belarus, Nezavisimosti Ave., 66, 220072 Minsk, Belarus; 4 School of Environmental Engineering and Chemistry, Luoyang Institute of Science and Technology, Luoyang 471000, China

**Keywords:** formic acid dehydrogenation, single-atom catalysts, DFT, graphitic carbon nitride

## Abstract

The rational development of single-atom catalysts (SACs) for selective formic acid dehydrogenation (FAD) requires an atomic-scale understanding of metal–support interactions and electronic modulation. In this study, spin-polarized density functional theory (DFT) calculations were performed to systematically examine platinum-group SACs anchored on graphitic carbon nitride (g-C_3_N_4_). The findings reveal that Pd and Au SACs exhibit superior selectivity toward the dehydrogenation pathway, lowering the free energy barrier by 1.42 eV and 1.39 eV, respectively, compared to the competing dehydration route. Conversely, Rh SACs demonstrate limited selectivity due to nearly equivalent energy barriers for both reaction pathways. Stability assessments indicate robust metal–support interactions driven by d–p orbital hybridization, while a linear correlation is established between the d-band center position relative to the Fermi level and catalytic selectivity. Additionally, charge transfer (ranging from 0.029 to 0.467 e) substantially modulates the electronic structure of the active sites. These insights define a key electronic descriptor for SAC design and offer a mechanistic framework for optimizing selective hydrogen production.

## 1. Introduction

Energy is the cornerstone of modern civilization, driving both economic development and technological advancement. However, the extensive reliance on fossil fuels has led to an energy crisis and severe environmental degradation, necessitating the development of sustainable alternatives [1]. Hydrogen, with its high gravimetric energy density, abundance, and environmentally benign combustion byproduct (water), has emerged as a promising energy carrier [2]. Despite these advantages, practical limitations in hydrogen storage and transportation hinder its widespread implementation [3].

Conventional storage methods, including high-pressure and cryogenic systems, pose significant safety risks [4]. In contrast, chemical hydrogen storage via liquid organic hydrogen carriers (LOHCs) offers a safer and more efficient alternative [5], enabling reversible hydrogen release under mild catalytic conditions. Among LOHCs, formic acid is particularly attractive due to its high hydrogen content (4.4 wt%), ambient stability, low toxicity, and non-flammability [6]. Furthermore, formic acid can be synthesized via photocatalytic or electrocatalytic CO_2_ reduction [7], contributing to a sustainable carbon-neutral cycle.

Formic acid decomposition follows two competing pathways [8]: dehydrogenation (HCOOH → H_2_ + CO_2_, ΔG = −32.8 kJ/mol) and dehydration (HCOOH → H_2_O + CO, ΔG = −14.9 kJ/mol). Selectively promoting the dehydrogenation route is critical for high-purity hydrogen production, as CO from the dehydration pathway poisons Pt-based formic acid dehydrogenation (FAD) catalysts [9]. This challenge has driven extensive research efforts to develop catalysts that achieve near-complete dehydrogenation under mild conditions.

Since Coffey et al. first reported the catalytic dehydrogenation of formic acid in the 1960s [10], a multitude of numerous homogeneous catalysts, particularly those based on Ir and Ru, have demonstrated remarkable activity in this process. However, these systems suffer from drawbacks such as high ligand costs, poor stability, and complex recovery processes, limiting their large-scale application [11]. In contrast, heterogeneous catalysts, particularly those based on noble metals such as Au, Pt, and Pd, offer advantages in recyclability and scalability [12,13]. However, each metal presents challenges: Pd is susceptible to CO poisoning, Au exhibits low intrinsic activity at low temperatures, and non-precious metals, while stable, generally lack selectivity. To enhance catalytic performance, strategies such as alloying (e.g., Ag, Cu, Cr, Mn, Co) [14] and support engineering (e.g., activated carbon, carbon nanotubes, metal–organic frameworks, TiO_2_) [15] have been explored. These approaches aim to optimize noble metal utilization while balancing activity and stability. However, challenges such as structural degradation and mass transport limitations persist [16]. The scarcity and cost of noble metals have further spurred interest in non-precious metal catalysts, including Co, Ni, and Cu-based systems [17]. While these catalysts demonstrate long-term stability, they often exhibit poor selectivity, sluggish kinetics, and a tendency toward dehydration side reactions [18]. Consequently, the development of efficient formic acid dehydrogenation (FAD) catalysts remains an ongoing challenge.

Recent advancements in single-atom catalysts (SACs) have shown significant promise by maximizing atomic efficiency and enhancing selectivity. Bulusev et al. [19] synthesized single Pd atoms supported on N-doped carbon, achieving excellent catalytic stability and activity at 60 °C. Density functional theory (DFT) calculations indicated that Pd atoms preferentially bind to pyridinic N-edge sites, enhancing catalytic performance. Han et al. [20] developed Pt-modified Te nanowires that achieved 100% selective hydrogen production, while Yang et al. [20] demonstrated that single-atom Pt/TiN catalysts effectively modulate the activity and selectivity of small organic molecules, favoring the direct dehydrogenation pathway in formic acid decomposition. Additionally, Shi et al. [20] reported a non-precious metal Co-based SAC with a hydrogen production rate of 1403.8 mL g⁻^1^ h⁻^1^, which is 15 times higher than that of commercial Pd/C catalysts.

Despite these advances, the high surface energy of single atoms and atomic clusters often leads to aggregation, resulting in catalyst deactivation. Thus, the selection of appropriate support material is crucial for stabilizing isolated metal atoms and preventing agglomeration. Among potential supports, graphitic carbon nitride (g-C_3_N_4_) has emerged as an excellent candidate due to its structural stability, unique electronic properties, and high porosity, which facilitate strong metal–support interactions [21]. SACs based on Pt-group metals (Pt, Pd, Ru) [22] have demonstrated remarkable catalytic activity and H_2_ selectivity, attributed to their electronic structure and well-defined metal–support interactions.

In this study, we investigate Pt-group SACs (M = Ru, Rh, Pd, Ag, Ir, Pt, Au) anchored on g-C_3_N_4_ using spin-polarized DFT calculations. By correlating d-band center positions, charge redistribution, and reaction energetics, we identify Pd and Au SACs as promising catalysts that favor dehydrogenation over dehydration, thereby suppressing CO formation. This work not only reveals a linear correlation relationship between the d-band center and selectivity but also provides valuable insights for the rational design of efficient SACs for hydrogen production.

## 2. Methods

Density functional theory (DFT) calculations were carried out using the hybrid Gaussian and plane wave (GPW) method [23] within the QUICKSTEP module of the CP2K 2023.1 package [24]. We utilized the double-zeta valence plus polarization (DZVP-MOLOPT-SR-GTH) basis set along with the Goedecker–Teter–Hutter (GTH) pseudopotential [25,26,27] for calculation. Electron density was expanded in an auxiliary plane wave basis set with a cutoff energy of 500 Ry. Exchange–correlation interactions were described using the spin-polarized Perdew–Burke–Ernzerhof (PBE) functional [28] under the generalized gradient approximation (GGA), with dispersion corrections applied via the DFT-D3 method of Grimme et al. [29].

The Kohn–Sham equations were solved using conventional matrix diagonalization, with Fermi–Dirac smearing (electronic temperature: 300 K) applied to enhance convergence. To improve computational efficiency, diagonalization routines from the ELPA library were employed [29]. Geometry optimizations were performed with energy and force convergence thresholds of 1.0 × 10^−6^ eV and 3.0 × 10⁻^4^ eV Å^−1^, respectively, while static calculations employed a tighter convergence criterion of 1.0 × 10^−8^ eV.

Brillouin zone sampling was achieved using a 3 × 3 × 1 Monkhorst-Pack k-point mesh for both g-C_3_N_4_ and M@g-C_3_N_4_ SACs. A 20 Å vacuum layer was introduced to eliminate spurious periodic interactions. Thermodynamic corrections and density of states (DOS) analyses were conducted using the Shermo v.2.6 [30] and Multiwfn v.3.8 [31] software packages.

## 3. Results and Discussion

### 3.1. Structure Stability and Metal–Support Interactions

Pt-group metals were chosen for their well-documented catalytic performance in formic acid dehydrogenation (FAD). The g-C_3_N_4_ substrate, consisting of 24 carbon and 32 nitrogen atoms, serves as an ideal optimal framework for single-atom anchoring. Previous studies [32] have identified the hollow site as the most thermodynamically favorable position for metal incorporation. Upon adsorption, a stable coordination environment is established between the metal atom and surrounding nitrogen atoms, effectively stabilizing the M@g-C_3_N_4_ SACs.

**Figure 1:**
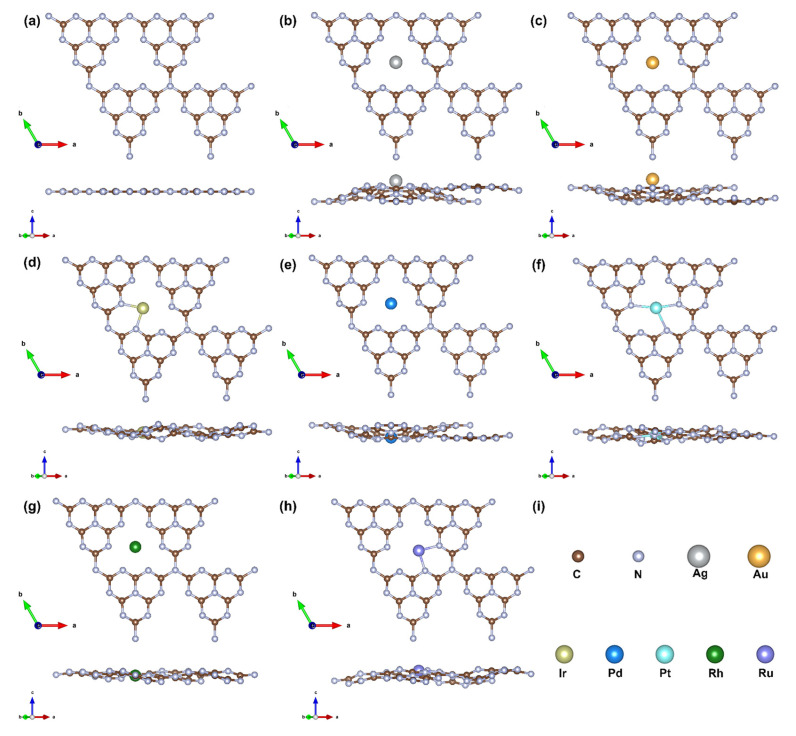
Atomic structure of the ground state of single-atom catalysts: (**a**) g-C_3_N_4_, (**b**) Ag@g-C_3_N_4_, (**c**) Au@g-C_3_N_4_, (**d**) Ir@g-C_3_N_4_, (**e**) Pd@g-C_3_N_4_, (**f**) Pt@g-C_3_N_4_, (**g**) Rh@g-C_3_N_4_, (**h**) Ru@g-C_3_N_4_. (**i**) The colors represent various elements.

Figure 1 illustrates the atomic structures of pristine g-C_3_N_4_ and various M@g-C_3_N_4_ SACs. While the undoped substrate retains a planar structure, metal doping induces localized distortions. Notably, Ag, Au, Pd, and Rh occupy nearly central positions within the g-C_3_N_4_ cavity, indicating symmetric coordination with the six surrounding nitrogen atoms. In contrast, Ir, Pt, and Ru exhibit off-center displacements, with Ir and Ru preferentially coordinating with two nitrogen atoms, whereas Pt binds to three nitrogen atoms, reflecting distinct metal–support interactions. The most significant factor contributing to this trend in M@g-C_3_N_4_ structures is the size of the cationic metal atoms [33]. The ionic radius of early-transition metal atoms (such as Ru in the fifth period, Ir and Pt in the sixth period) is larger than that of late-transition metal atoms (e.g., Rh, Pd, and Ag in the fifth period; Au in the sixth period). To accommodate these larger early-transition metal atoms ions, flexible g-C_3_N_4_ undergoes structural distortions.

The stability of SACs is crucial for their catalytic performance. To evaluate their thermodynamic stability, the binding energy (*E_b_*) of each SAC was computed as follows [34]:

(1) $$E_{b}=E_{M@g-C_{3}N_{4}}-E_{M}-E_{g-C_{3}N_{4}}$$

where *E_M@g-C3N4_*, *E_M_*, and *E_g-C3N4_* represent the total energies of the SACs system, isolated metal atom, and pristine g-C_3_N_4_ substrate, respectively. A negative binding energy confirms the thermodynamic stability of M@g-C_3_N_4_ SACs, with more negative values, with the magnitude of these energies reflecting the strength of the metal–support interactions, indicating greater structural stability.

**Figure 2:**
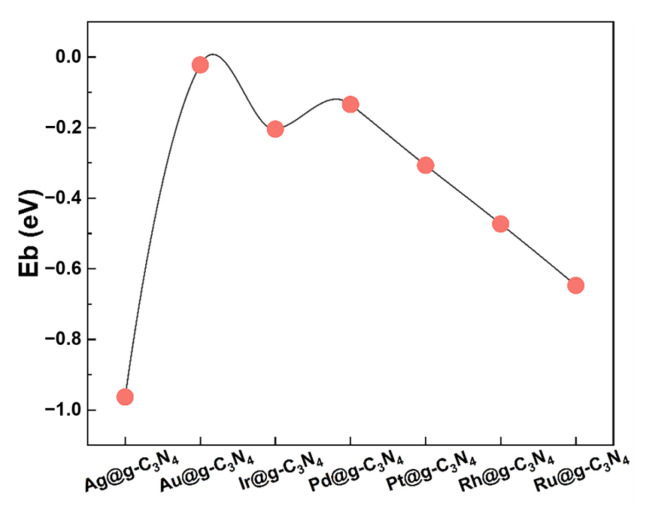
Binding energy of M@g-C_3_N_4_ SACs.

As shown in Figure 2, all SACs exhibit negative binding energies, reinforcing their thermodynamic feasibility. The stability trend is as follows: Ag > Ru > Rh > Pt > Ir > Pd > Au. As illustrated in Figure S1, the d orbitals of metal atoms in M@g-C_3_N_4_ SACs exhibit strong electronic interactions with the p orbitals of C and N in the g-C_3_N_4_ framework [35]. These interactions contribute to the remarkable stability of M@g-C_3_N_4_ SACs and serve as a fundamental basis for understanding their catalytic performance in FAD reactions.

### 3.2. Adsorption Behavior and Pathways for FAD

The adsorption behavior of formic acid and its key intermediates (HCOO, COOH, and CO) on the M@g-C_3_N_4_ SACs surfaces was evaluated to understand the reaction mechanism. The adsorption energy is defined as [36]

(2) $$E_{ad}=E_{M@g-C_{3}N_{4}+x}-E_{M@g-C_{3}N_{4}}-E_{x}$$

where *E_ad_*, *E_M@g-C3N4+x_*, *E_M@g-C3N4_*, and *E_x_* correspond to the adsorption energy, total energy of the SACs with an adsorbate, total energy of pristine SAC, and the energy of gas-phase adsorbates, respectively.

**Figure 3:**
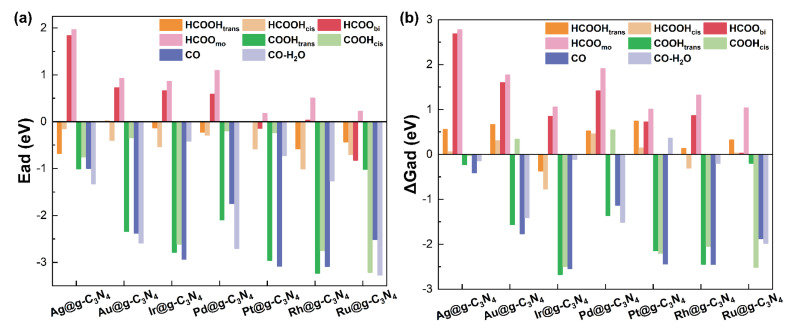
(**a**) Adsorption energy and (**b**) adsorption free energy of reactants and key intermediates on M@g-C_3_N_4_ SACs surface.

We first computed the adsorption energies (Figure 3a and Table S1) of HCOOH in both cis (HCOOH_cis_) and trans (HCOOH_trans_) configurations, as well as its dehydrogenation and dehydration intermediates. These include the bidentate (HCOO_bi_) and monodentate (HCOO_mo_) formats of formate, which have been identified through in situ infrared spectroscopy [37], along with COOH (cis and trans) [38], and the final CO product. Our calculations reveal that HCOOH adsorption is exothermic across all SACs, with *E_ad_* values ranging from −0.01 to −1.01 eV, suggesting its favorable interaction with the catalyst surface. The bidentate formate (HCOO_bi_) intermediate preferentially adsorbs on Pt and Ru SACs, whereas its monodentate counterpart (HCOO_mo_) exhibits weaker binding across all SACs, indicating that monodentate HCOO may be a crucial reaction intermediate in formic acid decomposition. Similarly, COOH adsorption is exothermic (−0.20 to −3.23 eV), while CO strongly adsorbs to all SACs, particularly on Pt (−3.08 eV) and Rh (−3.09 eV), where *E_ad_* values exceed −3.00 eV. To further refine our thermodynamic analysis, we incorporated zero-point energy and vibrational contributions to compute the adsorption free energy (ΔG), as shown in Figure 3b and Table S2. Notably, while ΔG for CO adsorption remained negative across all SACs, the inclusion of water molecules led to positive ΔG values on Ir and Pt SACs, indicating potential CO desorption in aqueous environments.

**Scheme 1:**
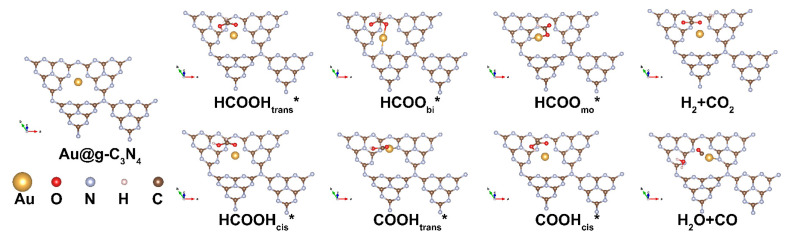
Dehydrogenation and dehydration pathways of formic acid on Au@g-C_3_N_4_ surface, where the * represents molecules adsorbed on the surface of the catalyst.

To elucidate the catalytic mechanism, we investigated two competing pathways for formic acid decomposition: dehydrogenation (Path 1, via the HCOO intermediate) and dehydration (Path 2, via the COOH intermediate), as illustrated in Scheme 1.

Path 1 (dehydrogenation): Formic acid initially adsorbs onto the SAC surface in a trans configuration, undergoes O–H bond cleavage to form bidentate HCOO, and then transitions to a monodentate structure before C–H bond cleavage releases H_2_ and CO_2_. Path 2 (dehydration): The reaction proceeds through cis-configured formic acid, followed by C–H bond cleavage yielding trans-configured COOH, which subsequently transitions to a cis configuration before undergoing O–O bond cleavage to generate H_2_O and CO.

### 3.3. Catalytic Activity and Selectivity

**Figure 4:**
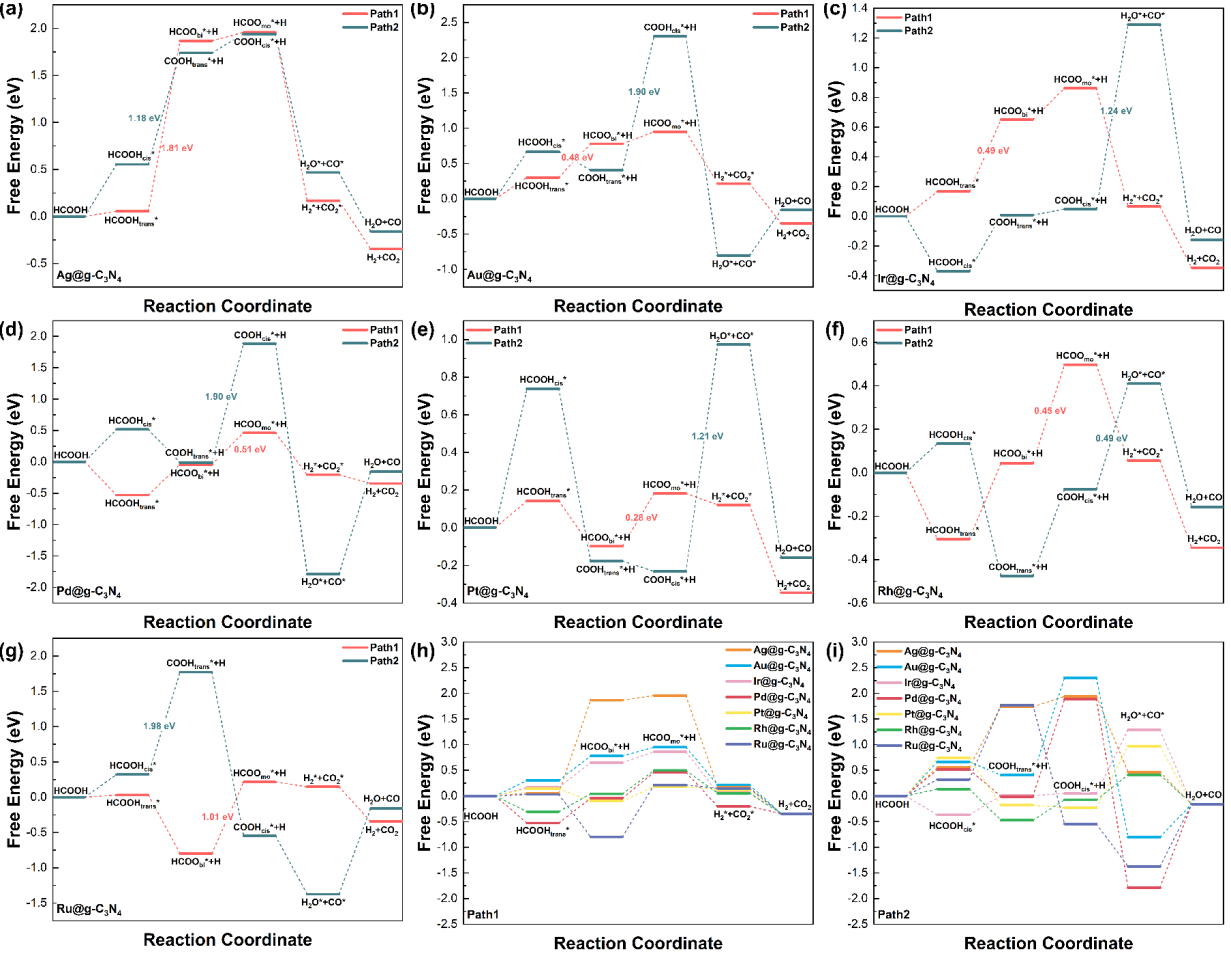
Free energy diagram for the decomposition of formic acid via dehydrogenation (Path1) and dehydration (Path2) pathways on (**a**) Ag@g-C_3_N_4_ SAC, (**b**) Au@g-C_3_N_4_ SAC, (**c**) Ir@g-C_3_N_4_ SAC, (**d**) Pd@g-C_3_N_4_ SAC, (**e**) Pt@g-C_3_N_4_ SAC, (**f**) Rh@g-C_3_N_4_ SAC, (**g**) Ru@g-C_3_N_4_ SAC, and the free energy for (**h**) Path1, and (**i**) Path2 of M@g-C_3_N_4_ SACs, where the * represents molecules adsorbed on the surface of the catalyst.

The free energy profiles (Figure 4) highlight significant differences between these pathways on different SACs. Among the various SACs, the maximum elementary step free energy barriers for dehydrogenation were 1.81 eV (Ag), 0.48 eV (Au), 0.49 eV (Ir), 0.51 eV (Pd), 0.28 eV (Pt), 0.45 eV (Rh), and 1.01 eV (Ru), while the free energy barriers for dehydration were 1.18 eV (Ag), 1.90 eV (Au), 1.24 eV (Ir),1.90 eV (Pd), 1.21 eV (Pt), 0.49 eV (Rh), and 1.98 eV (Ru).

As shown in Figure 4, the rate-determining step (RDS) in the dehydrogenation pathway of formic acid involves O–H bond cleavage in HCOOH for Ag, Au, and Ir SACs, whereas for Pd, Pt, Rh, and Ru SACs, the RDS corresponds to a configurational transformation of the adsorbed HCOO intermediate. In the competing dehydration pathway, the RDS is assigned to C–H bond cleavage in formic acid for Ag and Ru SACs, to an adsorption configuration transition of COOH for Au and Pd SACs, and to O–C bond cleavage in COOH for Ir, Pt, and Rh SACs.

The Au and Pd SACs display notably lower energy barriers for dehydrogenation compared to dehydration, with reductions of approximately 1.42 eV for Au and 1.39 eV for Pd. This energetic preference strongly favors the production of H_2_ over the formation of CO, which is a critical advantage considering CO’s detrimental effect on catalyst longevity, making them promising candidates for selective H_2_ generation, as shown in Table S3. In contrast, Rh SACs exhibit similar energy barriers for both pathways, leading to non-selective behavior that may compromise the purity of the hydrogen produced.

**Figure 5:**
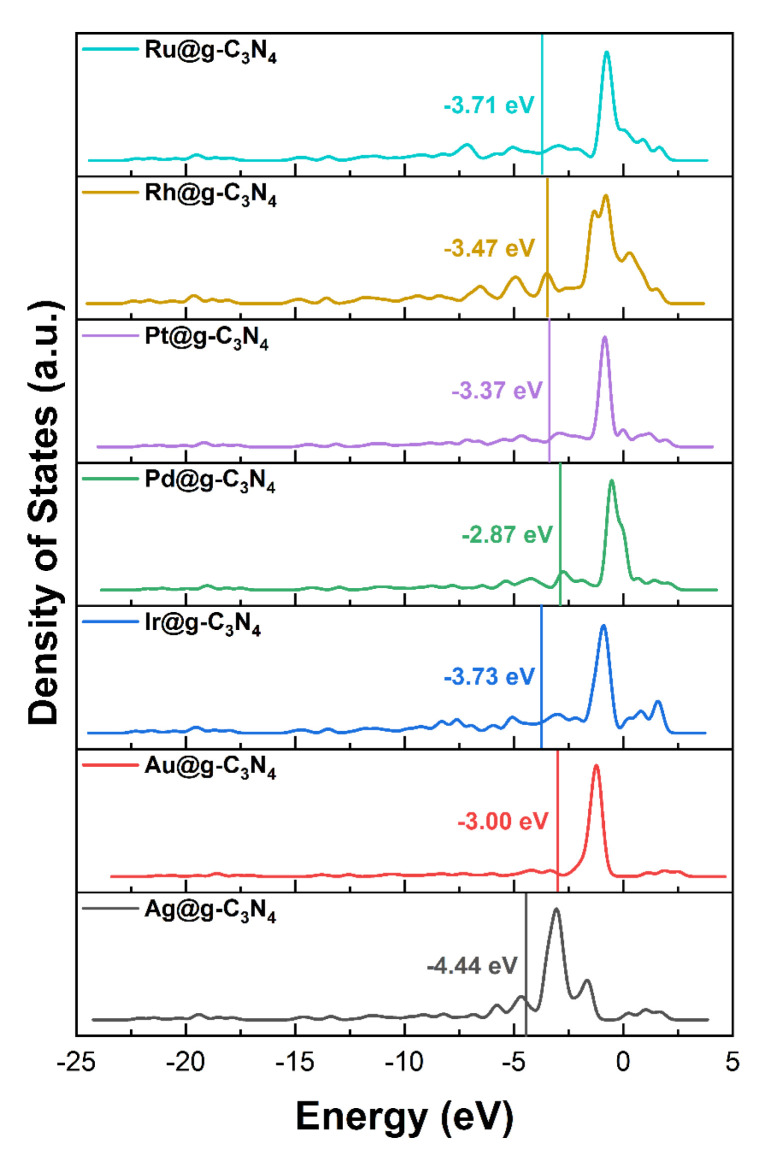
The d orbital density of state and d-band center of M@g-C_3_N_4_.

To understand the origin of Pd SAC selectivity, we analyzed the density of states (DOS) and d-band center of M@g-C_3_N_4_ SACs (Figure 5). The sharp, well-defined d-orbital peaks suggest near-free-electron behavior [39].

**Figure 6:**
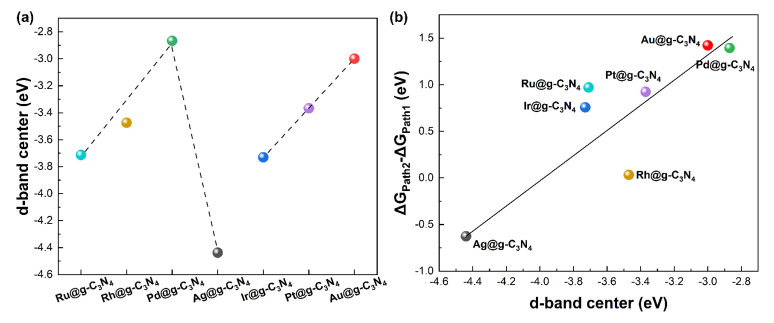
Relationship between the d band center of (**a**) atomic number and (**b**) formic acid decomposition selectivity.

As shown in Figure 6a, the d-band center exhibits a volcano-type relationship with atomic number for period IV metal SACs, whereas it shifts monotonically positive for period V metals. More importantly, as shown in Figure 6b, d-band center correlates linearly with formic acid decomposition selectivity, which indicates the SACs with a d-band center closer to the Fermi level exhibit higher selectivity toward dehydrogenation. This trend suggests that the electronic structure can be used as a predictive descriptor for catalyst performance. The proximity of the d-band center to the Fermi level directly influences the strength of the intermediate adsorption reaction on the catalyst [40]. Consequently, tuning the electronic structure can effectively alter the reactivity and selectivity of the catalytic material.

**Figure 7:**
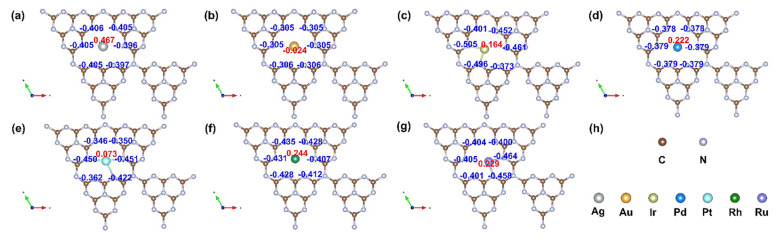
The Mulliken atomic charge distribution of (**a**) Ag@g-C_3_N_4_, (**b**) Au@g-C_3_N_4_, (**c**) Ir@g-C_3_N_4_, (**d**) Pd@g-C_3_N_4_, (**e**) Pt@g-C_3_N_4_, (**f**) Rh@g-C_3_N_4_, (**g**) Ru@g-C_3_N_4_. (**h**) The colors represent various elements.

Further, as shown in Figure 7, the Mulliken charge analysis indicates that charge transfer occurs from the metal atom to the g-C_3_N_4_ support, with values of 0.467 (Ag), −0.024 (Au), 0.164 (Ir), 0.222 (Pd), 0.073 (Pt), 0.244 (Rh), and 0.029 e (Ru). This redistribution of charge modifies the electronic environment at the active sites, influencing both the adsorption strengths of intermediates and the overall reaction kinetics. The modulation of electronic properties through metal–support interactions provides a clear pathway for tailoring catalyst performance, emphasizing the potential of electronic descriptors in guiding the rational design of next-generation SACs.

Overall, our results demonstrate that the careful selection of metal species and support material can effectively steer the reaction pathway toward selective dehydrogenation of formic acid. The combined insights from structural, energetic, and electronic analyses pave the way for designing catalysts that optimize hydrogen production while minimizing undesirable side reactions. Future work will focus on experimental validation and the exploration of bimetallic systems to further enhance catalytic performance.

## 4. Conclusions

This study elucidates the structure–activity relationships governing Pt-group SACs supported on g-C_3_N_4_ for formic acid dehydrogenation. Pd and Au SACs emerge as highly selective catalysts, achieving dehydrogenation barriers below 0.51 eV while suppressing CO formation through unfavorable dehydration pathways. Rh SACs, despite high activity, suffer from poor selectivity due to nearly equivalent barriers for both reaction routes. Moreover, Pd and Au emerge as promising catalytic candidates for hydrogen generation from formic acid, owing to their relatively lower cost compared to Rh. The thermodynamic stability of SACs is attributed to strong metal–support interactions, evidenced by negative binding energies and d–p orbital hybridization. A linear correlation between the d-band center position and selectivity highlights the critical role of electronic structure in catalytic performance. Charge redistribution (0.029–0.467 e) further fine-tunes the metal centers’ reactivity. These results underscore the potential of g-C_3_N_4_-supported SACs for efficient hydrogen production and provide a roadmap for optimizing catalyst design through electronic modulation and support engineering. Future work should focus on experimental validation and exploring synergistic effects of bimetallic SACs.

## Data Availability

The original contributions presented in the study are included in the article/Supplementary Materials, further inquiries can be directed to the corresponding authors.

## Supplementary Materials

The following supporting information can be downloaded at: https://www.mdpi.com/article/10.3390/ma18102328/s1, Figure S1: The density of states of p orbital of CN the d orbital of a metal atom in M@g-C_3_N_4_; Table S1: Adsorption energy of reactants and key intermediates on M@g-C_3_N_4_ SACs surface; Table S2: Adsorption free energy of reactants and key intermediates on M@g-C_3_N_4_ SACs surface, and Table S3: The d-band center, dehydrogenation reaction (Path1) free energy barrier, dehydration reaction (Path2) free energy barrier, formic acid decomposition selectivity and binding energy of M@g-C_3_N_4_ SACs.

## Abbreviations

The following abbreviations are used in this manuscript:

DFT	Density Functional Theory
SACs	Single Atom Catalysts
FAD	Formic Acid Dehydrogenation
g-C_3_N_4_	graphitic carbon nitride

## References

[1] Wang C.R., Stansberry J.M., Mukundan R., Chang H.-M.J., Kulkarni D., Park A.M., Plymill A.B., Firas N.M., Liu C.P., Lang J.T. (2025). Proton exchange membrane (PEM) water electrolysis: Cell-level considerations for gigawatt-scale deployment. Chem. Rev..

[2] Ahmad S., Ullah A., Samreen A., Qasim M., Nawaz K., Ahmad W., Alnaser A., Kannan A.M., Egilmez M. (2024). Hydrogen production, storage, transportation and utilization for energy sector: A current status review. J. Energy Storage.

[3] Squadrito G., Maggio G., Nicita A. (2023). The green hydrogen revolution. Renew. Energy.

[4] Zhu Q.-L., Xu Q. (2015). Liquid organic and inorganic chemical hydrides for high-capacity hydrogen storage. Energy Environ. Sci..

[5] Yadav M., Xu Q. (2012). Liquid-phase chemical hydrogen storage materials. Energy Environ. Sci..

[6] Chen B.W.J., Mavrikakis M. (2020). Formic acid: A hydrogen-bonding cocatalyst for formate decomposition. ACS Catal..

[7] Martin C., Quintanilla A., Vega G., Casas J.A. (2022). Formic acid-to-hydrogen on pd/AC catalysts: Kinetic study with catalytic deactivation. Appl. Catal. B.

[8] Dai H., Xia B., Wen L., Du C., Su J., Luo W., Cheng G. (2015). Synergistic catalysis of AgPd@ZIF-8 on dehydrogenation of formic acid. Appl. Catal. B.

[9] Choi B.-S., Song J., Song M., Goo B.S., Lee Y.W., Kim Y., Yang H., Han S.W. (2019). Core–shell engineering of pd–Ag bimetallic catalysts for efficient hydrogen production from formic acid decomposition. ACS Catal..

[10] Coffey R.S. (1967). A catalyst for the homogeneous hydrogenation of aldehydes under mild conditions. Chem. Commun..

[11] Boddien A., Junge H. (2011). Acidic ideas for hydrogen storage. Nat. Nanotechnol..

[12] Huang Y., Zhou X., Yin M., Liu C., Xing W. (2010). Novel PdAu@Au/C core−shell catalyst: Superior activity and selectivity in formic acid decomposition for hydrogen generation. Chem. Mater..

[13] Yin R., Jiang B., Guo H. (2022). Mechanism and dynamics of CO_2_ formation in formic acid decomposition on Pt surfaces. ACS Catal..

[14] Sun Q., Wang N., Xu Q., Yu J. (2020). Nanopore-supported metal nanocatalysts for efficient hydrogen generation from liquid-phase chemical hydrogen storage materials. Adv. Mater..

[15] Li Z., Yang X., Tsumori N., Liu Z., Himeda Y., Autrey T., Xu Q. (2017). Tandem nitrogen functionalization of porous carbon: Toward immobilizing highly active palladium nanoclusters for dehydrogenation of formic acid. ACS Catal..

[16] Yu W.-Y., Mullen G.M., Flaherty D.W., Mullins C.B. (2014). Selective hydrogen production from formic acid decomposition on pd–Au bimetallic surfaces. J. Am. Chem. Soc..

[17] Onishi N., Iguchi M., Yang X., Kanega R., Kawanami H., Xu Q., Himeda Y. (2019). Development of effective catalysts for hydrogen storage technology using formic acid. Adv. Energy Mater..

[18] Nie R., Tao Y., Nie Y., Lu T., Wang J., Zhang Y., Lu X., Xu C.C. (2021). Recent Advances in Catalytic Transfer Hydrogenation with Formic Acid over Heterogeneous Transition Metal Catalysts. ACS Catal..

[19] Bulushev D.A., Zacharska M., Shlyakhova E.V., Chuvilin A.L., Guo Y., Beloshapkin S., Okotrub A.V., Bulusheva L.G. (2016). Single isolated Pd^2+^ cations supported on N-doped carbon as active sites for hydrogen production from formic acid decomposition. ACS Catal..

[20] Han L., Zhang L., Wu H., Zu H., Cui P., Guo J., Guo R., Ye J., Zhu J., Zheng X. (2019). Anchoring Pt single atoms on Te nanowires for plasmon-enhanced dehydrogenation of formic acid at room temperature. Adv. Sci..

[21] Rocha G.F.S.R., da Silva M.A.R., Rogolino A., Diab G.A.A., Noleto L.F.G., Antonietti M., Teixeira I.F. (2023). Carbon nitride based materials: More than just a support for single-atom catalysis. Chem. Soc. Rev..

[22] Liu X., Su P., Chen Y., Zhu B., Zhang S., Huang W. (2018). g-C3N4 supported metal (pd, Ag, Pt) catalysts for hydrogen-production from formic acid. New J. Chem..

[23] Lippert G., Hutter J., Parrinello M. (1997). A hybrid gaussian and plane wave density functional scheme. Mol. Phys..

[24] Kühne T.D., Iannuzzi M., Del Ben M., Rybkin V.V., Seewald P., Stein F., Laino T., Khaliullin R.Z., Schütt O., Schiffmann F. (2020). CP2K: An electronic structure and molecular dynamics software package—Quickstep: Efficient and accurate electronic structure calculations. J. Chem. Phys..

[25] VandeVondele J., Hutter J. (2007). Gaussian basis sets for accurate calculations on molecular systems in gas and condensed phases. J. Chem. Phys..

[26] Krack M. (2005). Pseudopotentials for H to Kr optimized for gradient-corrected exchange-correlation functionals. Theor. Chem. Acc..

[27] Goedecker S., Teter M., Hutter J. (1996). Separable dual-space gaussian pseudopotentials. Phys. Rev. B.

[28] Perdew J.P., Burke K., Ernzerhof M. (1996). Generalized gradient approximation made simple. Phys. Rev. Lett..

[29] Grimme S., Antony J., Ehrlich S., Krieg H. (2010). A consistent and accurate ab initio parametrization of density functional dispersion correction (DFT-D) for the 94 elements H-Pu. J. Chem. Phys..

[30] Lu T., Chen Q. (2021). Shermo: A general code for calculating molecular thermochemistry properties. Comput. Theor. Chem..

[31] Lu T., Chen F. (2012). Multiwfn: A multifunctional wavefunction analyzer. J. Comput. Chem..

[32] Zheng Y., Jiao Y., Zhu Y., Cai Q., Vasileff A., Li L.H., Han Y., Chen Y., Qiao S.-Z. (2017). Molecule-level g-C_3_N_4_ coordinated transition metals as a new class of electrocatalysts for oxygen electrode reactions. J. Am. Chem. Soc..

[33] Ghosh D., Periyasamy G., Pandey B., Pati S.K. (2014). Computational studies on magnetism and the optical properties of transition metal embedded graphitic carbon nitride sheets. J. Mater. Chem. C.

[34] Jin T., Guo L., Tang Q., Wang J., Pan B., Wang C., Li Z., Chen F. (2022). Spontaneous formate oxidation on the 2D surface metal fluoride interface reconstructed from the AgPdF surface. J. Phys. Chem. C.

[35] Zhang N., Gao Y., Ma L., Wang Y., Huang L., Wei B., Xue Y., Zhu H., Jiang R. (2023). Single transition metal atom anchored on g-C_3_N_4_ as an electrocatalyst for nitrogen fixation: A computational study. Int. J. Hydrogen Energy.

[36] Jin T., Chen F., Guo L., Tang Q., Wang J., Pan B., Wu Y., Yu S. (2021). Pd_4_O_3_ subsurface oxide on pd(111) formed during oxygen adsorption-induced surface reconstruction and its activity toward formate oxidation reactions. J. Phys. Chem. C.

[37] Tang Y., Roberts C.A., Perkins R.T., Wachs I.E. (2016). Revisiting formic acid decomposition on metallic powder catalysts: Exploding the HCOOH decomposition volcano curve. Surf. Sci..

[38] Fingerhut J., Lecroart L., Schwarzer M., Hörandl S., Borodin D., Kandratsenka A., Kitsopoulos T.N., Auerbach D.J., Wodtke A.M. (2024). Identification of reaction intermediates in the decomposition of formic acid on pd. Faraday Discuss..

[39] Greiner M.T., Jones T.E., Beeg S., Zwiener L., Scherzer M., Girgsdies F., Piccinin S., Armbrüster M., Knop-Gericke A., Schlögl R. (2018). Free-atom-like d states in single-atom alloy catalysts. Nature Chem.

[40] Yang Y., Peltier C.R., Zeng R., Schimmenti R., Li Q., Huang X., Yan Z., Potsi G., Selhorst R., Lu X. (2022). Electrocatalysis in alkaline media and alkaline membrane-based energy technologies. Chem. Rev..

