# Supplementary material for: Theoretical Insights into Hydrogen Production from Formic Acid Catalyzed by Pt-Group Single-Atom Catalysts
Source: Materials (Basel). 2025 May 16;18(10):2328. doi: 10.3390/ma18102328 (PMC12112991; doi:10.3390/ma18102328)
Supplement: Supplementary file 1 [file materials-18-02328-s001.zip › materials-3604033-supplementary.pdf]

Supplementary Materials

Theoretical Insights into Hydrogen Production from Formic Acid Catalyzed by Pt-Group Single-Atom Catalysts

Tao Jin [1,*], Sen Liang [2], Jiahao Zhang [2], Yaru Li [2], Yukun Bai [1], Hangjin Wu [1], Ihar Razanau [3], Kunming Pan [1,2,*] and Fang Wang [1,4]

1 Longmen Laboratory, Luoyang 471000, China; wangfang1116@163.com (F.W.)
2 School of Materials Science and Engineering, Henan University of Science and Technology, Luoyang 471000, China
3 Scientific-Practical Materials Research Centre, National Academy of Sciences of Belarus, Nezavisimosti Ave., 66, 220072 Minsk, Belarus
4 School of Environmental Engineering and Chemistry, Luoyang Institute of Science and Technology, Luoyang 471000, China
* Correspondence: jint@longmenlab.com (T.J.); pankunming2008@haust.edu.cn (K.P.)

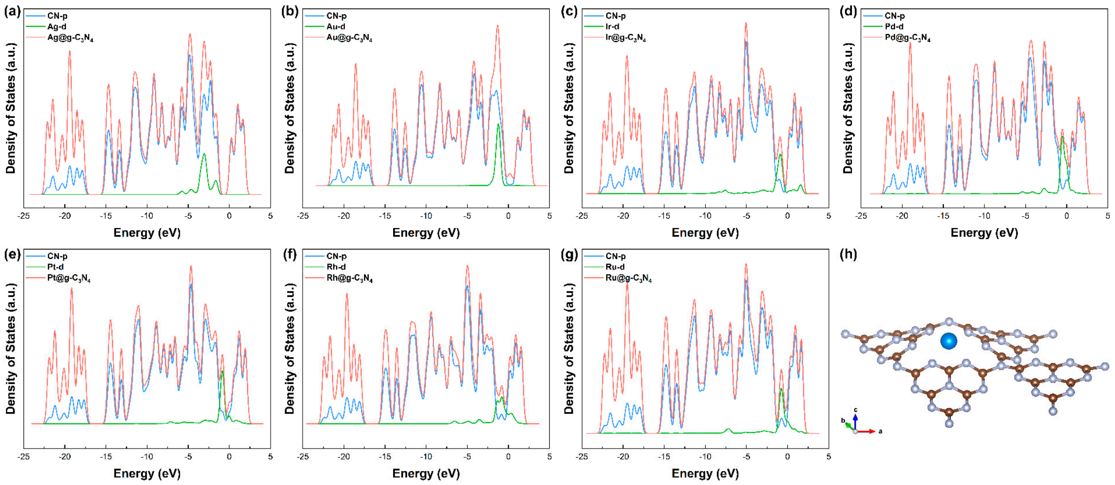

Figure S1. The density of states of p orbital of CN the d orbital of a metal atom in M@g-C_3N_4

Table S1. Adsorption energy of reactants and key intermediates on M@g-C_3N_4 SACs surface.

	Ead/eV							
	HCOOH trans	HCOOH cis	HCOO bi	HCOO mo	COOH trans	COOH cis	CO	CO+H_2O
Ag@g-C_3N_4	-0.68	-0.15	1.84	1.96	-1.00	-0.75	-1.00	-1.33
Au@g-C_3N_4	0.01	-0.40	0.72	0.92	-2.34	-0.35	-2.38	-2.59
Ir@g-C_3N_4	-0.13	-0.53	0.66	0.86	-2.79	-2.61	-2.93	-0.42
Pd@g-C_3N_4	-0.22	-0.29	0.59	1.09	-2.10	-0.20	-1.74	-2.70
Pt@g-C_3N_4	-0.01	-0.58	-0.14	0.18	-2.96	-0.24	-3.08	-0.73
Rh@g-C_3N_4	-0.58	-1.01	0.03	0.50	-3.23	-2.75	-3.09	-1.26
Ru@g-C3N4	-0.43	-0.70	-0.82	0.22	-1.02	-3.21	-2.51	-3.27

Table S2. Adsorption free energy of reactants and key intermediates on M@g-C_3N_4 SACs surface.

	Gad/eV							
	HCOOH trans	HCOOH cis	HCOO bi	HCOO mo	COOH trans	COOH cis	CO	CO+H_2O
Ag@g-C_3N_4	0.55	0.06	2.69	2.78	-0.23	-0.03	-0.41	-0.14
Au@g-C_3N_4	0.67	0.30	1.60	1.77	-1.56	0.34	-1.77	-1.41
Ir@g-C_3N_4	-0.37	-0.77	0.85	1.05	-2.67	-2.49	-2.54	-0.11
Pd@g-C_3N_4	0.52	0.46	1.41	1.91	-1.36	0.54	-1.14	-1.51
Pt@g-C_3N_4	0.74	0.14	0.72	1.00	-2.14	-2.20	-2.44	0.36
Rh@g-C_3N_4	0.13	-0.31	0.86	1.32	-2.44	-2.04	-2.45	-0.20
Ru@g-C3N4	0.32	0.03	0.02	1.04	-0.20	-2.51	-1.88	-1.98

Table S3. The d-band center, dehydrogenation reaction (Path1) free energy barrier, dehydration reaction (Path2) free energy barrier, formic acid decomposition selectivity and binding energy of M@g-C_3N_4 SACs.

	d-band center/eV	ΔG_{Path1}/eV	ΔG_{Path2}/eV	ΔG_{Path2}-ΔG_{Path1}/eV	E_b/eV
Ag@g-C_3N_4	-4.44	1.81	1.18	-0.63	-0.96
Au@g-C_3N_4	-3.00	0.48	1.90	1.42	-0.02
Ir@g-C_3N_4	-3.73	0.49	1.24	0.76	-0.20
Pd@g-C_3N_4	-2.87	0.51	1.90	1.39	-0.13
Pt@g-C_3N_4	-3.37	0.28	1.21	0.92	-0.31
Rh@g-C_3N_4	-3.47	0.45	0.49	0.03	-0.47
Ru@g-C_3N_4	-3.71	1.01	1.98	0.97	-0.65
